# Genito-Urinary Diseases—Circumcision1Delivered at the Erie County Hospital, Buffalo, N. Y., December, 1897.

**Published:** 1898-04

**Authors:** Ross G. Loop


### Clinical Lecture.

### GENITO-URINARY DISEASES—CIRCUMCISION.[1]

BY B. H. DAGGETT, M. D.
REPORTED BY ROSS G. LOOP, M. D.

CIRCUMCISION is a procedure older than history and is traced back into the mists of tradition and mythology. There is a legend that an Atlantean king ordered his entire army circumcised in order to escape a prevailing scourge. Bergman states that circumcision was at one time performed for the purpose of making an indestructible mark of servitude.[2] The Basanta, of Australia, do the circular operation and place the excised portion upon the middle finger of the left hand for a ring and many other savage tribes perform some one of the various operations of circumcision and associate with it elaborate ceremonies and curious observances. It was practised by the Egyptians six centuries before Christ. The uncircumcised were looked upon with disfavor in the V. century. It was common among the Arabs long before the days of Mohammed, who was himself circumcised, and who is reported to have said: "Circumcision is an ordinance for men and honorable in women." In Turkey, a tax was levied upon the uncircumcised, while in Rome it was placed upon the circumcised.

The command to Abraham was as follows: "This is my covenant, which ye shall keep between me and you, and thy seed after thee: every man child among you shall be circumcised, and ye shall circumcise the flesh of the fore-skin, and it shall be a covenant betwixt thee and me."

Paul was charged with disregarding the law, forsaking the teaching of Moses, and attempting to abolish circumcision. Sarah conceived after the circumcision of Abraham.

---

1. Delivered at the Erie County Hospital, Buffalo, N. Y., December, 1897.
2. Remondino, Circumcision.

Circumcision has been performed as a purely religious rite, a covenant and an offering, without any thought of sanitary benefit, and had its origin as a general practice with the foundation of the Hebraic creed, in the hills of Chaldea. It was designated as a law of perpetual obligation upon the Hebrew family, under penalty of excommunication. Remondino says : "There are Hebrews, however, who look upon the practice as an arbitrary rule of the remote past and claim that it is hygienic, that it has no ritual significance and that in modern times it has no purpose or use, except that relating to pathology.

Nearly every man child is born into the world phymosed and if he lives to old age uncircumcised goes out of it in the same condition. After a few years of growth and development, together with frequent ablutions, this state of affairs is usually satisfactorily adjusted by nature and all goes well, until, with declining age and abatement of the genital function, the wasting phallus again withdraws within its preputial folds and becomes redolent with urinary and smegmatic exhalations.

It is evident that there was more than a sanitary or ritual purpose in this practice, since the circumcised phallus was an emblem of civil and religious orthodoxy, the symbol of national honor, an object of worship and was grasped in the hand when executing the jurat of the period. It is claimed that the prepuce, like the climbing and auricular muscles and other functionless organs of the body, is a relic of man's sylvian and barbaric existence and which has not yet been eradicated in the process of evolution. During those primitive days the prepuce served to protect the glans from erosion by briar and bramble as men sped upon all fours through primeval forests or climbed the first friendly tree to escape his gastronomic enemy.

Ricord called the prepuce a useless bit of flesh ; Remondino said it has neither use nor beauty, and Puzy, of Liverpool, says it makes most excellent skin grafts.

Phymosis signifies to tie with a string and is applied to both the redundant, retractable and unretractable foreskin. The complications arising from phymosis are balanites, balano-posthitis ; adhesions ; hemorrhoids ; irritability and dilatation of the bladder, ureters and renal pelves ; retention and incontinence of urine ; hernia ; distortion of the glans and urethra ; sexual excitement ; spastic palsies ; pseudo hip-joint disease ; muscular incoördination ; cancer and herpes.[1] The dangers arising from this con-

1. Remondino, Circumcision.

dition and the imperative necessity of relief are, therefore, apparent.

Of the various operations for circumcision, the circular is by far the most common and is fully described in your text-books. This operation leaves the glans in an unprotected condition, which hardens the sensitive papillæ and causes atrophy of Tyson's glands. There is another procedure, which consists in slitting through the middle dorsal line from the orificial margin to the corona. This leaves a large lateral ear on either side, which is objected to by adults on esthetic grounds. Slitting the foreskin along the frenum was proposed by Celsus, revived by Cloquet, and condemned by Ricord and others. Remondino, in thin, narrow foreskins, recommends a combination of the dorsal and inferior slits. Dilatation and divulsion have been suggested, but have not received favor from the profession. Lallemand proposed transfixion of the prepuce on the dorsum and over the anfractus and from this point cutting away the flaps. McLeod proposed suturing before removing the prepuce. Gross favored the double slit. Van Buren and Keyes combine the dorsal slit with the Ricord-Bumstead operation. Ligation and the cautery have been used.

In the case before us today, I transfix the dorsal line of the prepuce about one-third of an inch from the orificial margin and carry the incision back to the coronal sulcus. On the inferior surface I make an incision from the prepucial orifice back for about half an inch. These incisions sever all constructing bands. Upon retracting the prepuce, these longitudinal incisions become transverse. The orifice of the prepuce is enlarged by the inferior slit. The lines of incision may be sutured with catgut, silk or silk-worm gut. Below, suture the skin and mucous membrane together, thus forming the new margin of the enlarged orifice. Above, stitch the edges of the skin together. The bulging of tissues at the ends of the new transverse incision, located behind the glans, will soon disappear. In dressing, Friar's balsam, applied to all except the apex of the glans, or collodion applied over the wounds, make an excellent protective. The foreskin must be held retracted until healing is complete.

This operation shortens and widens the prepuce; it sacrifices no tissue; it neither mutilates nor deforms; it reconstructs and creates a cosmetic, esthetic covering for the sensitive glans and its Tysonian brood.

